# Composition and cytotoxic and antioxidant activities of the oil of *Piper aequale* Vahl

**DOI:** 10.1186/s12944-016-0347-8

**Published:** 2016-10-07

**Authors:** Joyce Kelly R. da Silva, Laine C. Pinto, Rommel M. R. Burbano, Raquel C. Montenegro, Eloísa Helena A. Andrade, José Guilherme S. Maia

**Affiliations:** 1 Programa de Pós-Graduação em Biotecnologia, Universidade Federal do Pará, 66075-900 Belém, PA Brazil; 2 Laboratório de Citogenética Humana, Universidade Federal do Pará, 66075-900 Belém, PA Brazil; 3 Programa de Pós-Graduação em Química, Universidade Federal do Pará, 66075-900 Belém, PA Brazil; 4 Programa de Pós-Graduação em Recursos Naturais da Amazônia, Universidade Federal do Oeste do Pará, 68035-110 Santarém, PA Brazil

**Keywords:** Essential oil composition, Terpene compounds, Cytotoxic and antioxidant activities, Apoptosis

## Abstract

**Background:**

*Piper aequale* Vahl is a small shrub that grows in the shadow of large trees in the Carajás National Forest, Municipality of Parauapebas, Para state, Brazil. The local people have used the plant against rheumatism and inflammation.

**Methods:**

The essential oil of the aerial parts was extracted and analyzed by GC and GC-MS. The MTT colorimetric assay was used to measuring the cytotoxic activity of the oil against human cancer lines. The determination of antioxidant activity of the oil was conducted by DPPH radical scavenging assay.

**Results:**

The main constituents were δ-elemene (18.92 %), β-pinene (15.56 %), α-pinene (12.57 %), cubebol (7.20 %), β-atlantol (5.87 %), and bicyclogermacrene (5.51 %), totalizing 65.63 % of the oil. The oil displayed a strong in vitro cytotoxic activity against the human cancer cell lines HCT-116 (colon) and ACP03 (gastric) with IC_50_values of 8.69 μg/ml and 1.54 μg/ml, respectively. The oil has induced the apoptosis in a gastric cancer cells in all tested concentration (0.75–3.0 μg/ml), after 72 h of treatment, when compared to negative control (*p* < 0.001). Also, the oil showed a significant antioxidant activity (280.9 ± 22.2 mg TE/ml), when analyzed as Trolox equivalent, and a weak acetylcholinesterase inhibition, with a detection limit of 100 ng, when compared to the physostigmine standard (1.0 ng).

**Conclusion:**

The higher cell growth inhibition induced by the oil of *P. aequale* is probably due to its primary terpene compounds, which were previously reported in the proliferation inhibition, in stimulation of apoptosis and induction of cell cycle arrest in malignant cells.

## Background

Piperaceae comprises five genera and about 1400 species with a pantropical distribution [1]. *Piper* genus is the greater in the family and mainly distributed in the tropical and subtropical region of the world. It has been extensively investigated as the source of natural products with potential antifungal, antitumoral, antioxidant, antiplasmodial, and trypanocidal properties [2]. Moreover, *Piper* species are used to treat diseases, including jaundice, rheumatism, and neuralgia in the folk medicine of Asia and Pacific region [3]. *Piper aequale* Vahl, with several synonyms (http://www.tropicos.org/Name/25001129), is a shrub up 2.0 m with distribution throughout Central America and Northern of South America [3, 4]. In the Northeastern of Pará state, Brazil, the decoction of leaves is used in the treatment of rheumatism and inflammation.

*Piper* species have presented many classes of compounds, such as unsaturated amides, flavonoids, lignans, aristolactams, long and short chain esters, steroids, and alkaloids [5]. Moreover, the oils of *Piper in* the Brazilian Amazon have shown terpenoid and phenylpropanoid compounds as major constituents [6, 7]. Few works with *P. aequale* were published. The chemical studies have reported the presence of benzofuranoid neolignans and the mono- and sesquiterpenes compounds in the aerial parts [8, 9]; the insecticidal and larvicidal activity of the ethanolic extract [10]; as well the bactericidal activity of the ethanolic extract and essential oil [11].

The aim of this study was to identify the composition of the oil of *Piper aequale* and evaluate their cytotoxic, antioxidant and anticholinesterase properties.

## Methods

### Plant material

The aerial parts (leaves and thin stems) of *Piper aequale* were collected in the Carajás National Forest, Municipality of Parauapebas, Pará state, Brazil, February 2008. A voucher (MG 189272) was deposited in the herbarium of Emilio Goeldi Museum, Belém, Pará state, Brazil.

### Extraction of essential oil and composition analysis

The plant material was air dried, pulverized and submitted to hydrodistillation using a Clevenger-type apparatus (100 g, 3 h). The oil was dried over anhydrous sodium sulfate, and its percentage content was calculated based on the plant dry weight. The moisture content of the sample was calculated after phase separation in a Dean–Stark trap (5 g, 60 min), using toluene.

The oil analysis was carried on a GC-MS Thermo Focus DSQ II, under the following conditions: DB-5 ms (30 m × 0.25 mm, 0.25 mm film thickness) fused-silica capillary column; programmed temperature, 60–240 °C (3 °C/min); injector temperature, 250 °C; carrier gas helium, adjusted to a linear velocity of 32 cm/s (measured at 100 °C); injection type, splitless (2 μl of a 1:1000 hexane solution); split flow was adjusted to yield a 20:1 ratio; septum sweep was a constant 10 ml/min; EIMS, electron energy at 70 eV; temperature of the ion source and connection parts, 200 °C. The quantitative data regarding the volatile constituents were obtained by peak area normalization using a FOCUS GC/FID operated under similar conditions for the GC–MS, except the carrier gas, which was nitrogen. The retention index was calculated for all volatiles constituents using an *n*-alkane (C8-C30, Sigma–Aldrich) homologous series [12].

### Antioxidant assay

The antioxidant activity of the oil was evaluated by DPPH radical scavenging assay, using methodology adapted by us [13]. DPPH is a stable dark violet free radical with a maximum absorption at 517 nm. Trolox, a hydrophilic carboxylic acid derivative of α-tocopherol was employed to determine the radical scavenging activity, as a standard antioxidant. The radical-scavenging activity was expressed as milligrams of Trolox equivalent per milliliter of each sample (mgTE/ml).

### Acetylcholinesterase assay

The acetylcholinesterase (AChE) inhibition assay (by bioautography) is a rapid method and relatively free of disturbances by solvent [14]. The enzyme acetylcholinesterase (500 U) was dissolved in tris-hydrochloric acid buffer (pH 7.8) and stabilized by the addition of bovine serum albumin fraction V (0.1 %). TLC layers were spotted with the essentials oils in the range of 0.01 to 1000 ng/spot. The alkaloid physostigmine was used as positive control. The plates were then sprayed with the enzyme solution (3.33 U/ml), thoroughly dried and incubated at 37 °C for 20 min (moist atmosphere). Enzyme activity was detected by spraying with a solution consisting of 0.25 % of 1-naphtyl acetate in EtOH (5 ml) plus 0.25 % aqueous solution of Fast Blue B salt (20 ml). Potential acetylcholinesterase inhibitors appeared as clear zones on a purple colored background [15].

### Cytotoxicity against cancer cell lines

The MTT colorimetric assay was usedfor measuring cell viability [16]. The oil (0.4 to 25.0 μg/ml) was tested for cytotoxic activity against three cancer cell lines: HCT-116 (colon), SKMEL19 (melanoma) and ACP-03 (gastric). All cell lines were maintained in DMEM (Dulbecco’s Modified Eagle Medium) supplemented with 10 % fetal bovine serum, 2 mM glutamine, 100 U/ml penicillin, 100 μg/ml streptomycin at 37 °C with 5 % CO_2_. The oil was dissolved to obtain a concentration of 10 mg/ml with DMSO. The final concentration of DMSO in the culture medium was kept constant, below 0.1 % (v/v). The oil was incubated with the cells for 72 h. The negative control received the same amount of DMSO (0.001 % in the highest concentration). The cell viability was determined by reduction of the yellow dye 3-(4,5-dimethyl-2-thiazol)-2,5-diphenyl-2*H*-tetrazolium bromide (MTT) to a blue formazan product. The IC_50_’s values were calculated by nonlinear regression using GraphPad program (Intuitive Software for Science, San Diego, CA).

### Cell membrane disruption

The potential of the cell membrane lyses was evaluated by releasing hemoglobin from erythrocytes in the medium. The test was performed in 96-well plates using a 2 % mouse erythrocyte suspension in 0.85 % NaCl containing 10 mM CaCl_2_. The oils diluted as mentioned above were tested at 200 μg/ml. After incubation at room temperature for 1 h and centrifugation, the supernatant was removed, and the liberated hemoglobin was measured spectrophotometrically at 540 nm. DMSO was used as negative control and Triton X-100 (1 %) as the positive control [17].

### Evaluation of apoptosis and necrosis using differential fluorescent staining with ethidium bromide/acridine orange (EB/AO)

Cells were seeded into 12-well plates at a density of 1x10^4^cells/well, at 37 °C. After 24 h of incubation, cells were treated with the oil (0.75 μg/ml, 1.5 μg/ml and 3.0 μg/ml) for 72 h. Doxorubicin (1 μM) was used as a positive control. Cells were pelleted and resuspended in 20 μl phosphate-buffered saline (PBS). Afterward, 1 μl of an aqueous solution of EB/AO (100 μg/ml) was added and cells were observed under a fluorescence microscope (Olympus, Tokyo, Japan). Acridine orange intercalates into the DNA giving it a green appearance. Ethidium bromide also intercalates into DNA, making it appear orange, but it is only taken up by non-viable cells. Live cells with intact membranes have a uniform green color in their nuclei. Early apoptotic cells have chromatin condensation with bright green colored nuclei. Late apoptotic cells have bright orange areas of condensed chromatin in the nucleus that distinguish them from necrotic cells, which have a uniform orange color [18]. Three hundred cells were then classified as follows: live cells, apoptotic cells, and necrotic cells. The percentage of apoptotic cells and necrotic cells was then calculated. Experiments were performed in triplicate.

### Statistical analysis

Samples were assayed in triplicate, and the results are shown as means ± standard deviation. For antioxidant and cytotoxic screening, analysis of variance (ANOVA) was conducted by Tukey’s test and for the apoptosis evaluation, the Bonferroni test was used. The values between means were considered statistically significant at *p* < 0.05, using Graph Prism 5.0.

## Results and discussion

### Composition of the oil

The aerial parts (leaf and thin stems) of *P. aequale* provided an oil yield of 2.2 %,and GC and GC-MS analyzed its volatile constituents. The individual components were identified by comparison of both mass spectrum and GC retention data with those of authentic compounds existing in data system libraries [19, 20]. Thirty-six components were identified and comprised to 92.4 % of the total composition (Table 1). The oil was composed of sesquiterpenes hydrocarbons (42.5 %), followed by monoterpenes hydrocarbons (29.1 %) and oxygenated sesquiterpenes (20.9 %). The main constituents (above 5 %) were δ-elemene (19.0 %), β-pinene (15.6), α-pinene (12.6 %), cubebol (7.2 %), β-atlantol (5.9 %), and bicyclogermacrene (5.5 %).

**Table 1:** Oil composition (%) of *Piper aequale*

No.	Constituents	RI^a^	RI^b^	Oil %
01	α-Pinene	937	932	**12.6**
02	β-Pinene	980	974	**15.6**
03	Limonene	1031	1024	1.0
04	δ-Elemene	1333	1335	**19.0**
05	α-Cubebene	1345	1345	0.6
06	α-Ylangene	1365	1373	0.6
07	α-Copaene	1373	1374	0.9
08	β-Elemene	1382	1389	2.6
09	α-Gurjunene	1400	1409	0.1
10	β-Caryophyllene	1415	1417	1.3
11	β-Ylangene	1417	1419	0.4
12	γ-Elemene	1426	1434	1.6
13	Aromadendrene	1434	1439	0.1
14	*cis*-Muurola-3,5-diene	1445	1451	0.4
15	α-Humulene	1452	1452	0.7
16	*allo*-Aromadendrene	1456	1458	0.3
17	Iswharane	1464	1465	0.7
18	*cis*-Muurola-4(14),5-diene	1465	1465	0.6
19	Germacrene D	1477	1484	3.5
20	β-Selinene	1485	1489	0.2
21	*trans*-Muurola-4(14),5-diene	1487	1493	0.4
22	Bicyclogermacrene	1492	1500	**5.5**
23	α-Muurolene	1495	1500	0.7
24	Cubebol	1511	1514	**7.2**
25	δ-Cadinene	1515	1522	2.3
26	*trans*-Cadina-1,4-diene	1529	1533	0.2
27	α-Cadinene	1532	1537	0.1
28	Elemol	1545	1548	1.4
29	Germacrene B	1553	1559	0.1
30	Guaiol	1592	1600	0.9
31	β-Atlantol	1604	1608	**5.9**
32	*epi*-α-Muurolol	1639	1640	2.1
33	α-Muurolol	1642	1644	0.9
34	α-Cadinol	1650	1652	2.0
35	Selin-11-en-4-α-ol	1651	1658	0.1
36	Bulnesol	1662	1670	0.4
Monoterpene hydrocarbons				29.2
Sesquiterpenes hydrocarbons				42.9
Oxygenated sesquiterpenes				20.9
Total				93.0

^a^Retention indices calculated for all volatile constituents using a homologous series of *n*-alkanes

^b^Retention indices described in literature (Adams, 2007 [20])

The bold numbers represent the compounds with area percentage above 5%

The leaf oil of another specimen of *P. aequale* was reported to Monteverde, Costa Rica. It was composed by a high content of the monoterpene hydrocarbons α-pinene (39.3 %), sabinene (18.4 %) and limonene (6.7 %) [9]. Therefore, the oil with occurrence in Costa Rica differs somewhat from the oil that is being presented in this work, whose principal constituent is δ-elemene (19.0 %), a sesquiterpene hydrocarbon. However, as can be seen, the δ-elemene is followed by α-pinene and β-pinene, two monoterpene hydrocarbons. The isoforms β-, γ- and δ-elemene were identified as main compounds in other oils of *Piper* occurring at Brazilian Amazon, such as *P. aleyreanum* and *P. dilatatum* [7, 15]. Furthermore, the monoterpenes α-pinene and β-pinene have predominated in the oil of *P. anonifolium* [21]. The high content of bicyclogermacrene and germacrene D has also been found in oils of *P. arboreum* occurring on diverse Brazilian region [22–24].

### Antioxidant activity

The oil of *P. aequale* reduced DPPH radicals, and the absorbance of reaction mixture displayed a decreasing at 517 nm. After 30 min, the inhibition of DPPH radical was 25.9 ± 2.6 % corresponding to 280.9 ± 22.2 mg ET⁄mL. The antioxidant activity of essential oils rich in terpenoids, showing different protective effects on lipid oxidation, due their functional groups, have been reported [25]. The oil of *P. aleyreanum* with predomination of β-elemene (16.3 %) and δ-elemene (8.2 %) displayed potent antioxidant activity (412.2 ± 9.5 mg ET⁄mL) [15]. The oil of *Chimonanthus praecox*, a traditional Chinese plant which contains elemene, showed a marked antioxidant effect on scavenging peroxide and hydroxyl radicals [26]. Also, α-pinene and β-pinene presents in the oil of *P. aequale* as majority compounds, have exhibited around 50 % of the antioxidant activity of ascorbic acid, in the DPPH assay, and an EC_50_ value equivalent to BHT in a β-carotene⁄ linoleic acid system [27].

### Anticholinesterase activity

The oil of *P. aequale* showed a weak anticholinesterase activity, with a detection limit (DL) of 100 ng, Ie, about a hundred times less active than the standard physostigmine standard (DL = 1.0 ng). Previous studies reported that monoterpenes are most efficient on inhibition of AchE activity, in comparison with sesquiterpenes [28] α-Pinene showed anti-cholinesterase activity equivalent to galantamine (IC_50_ = 87.2 μM), used as positive control. However, its isomer β-pinene displayed a not significant activity [29]. Miyazawa and Yamafuji [29] explain that a small activity is due to the synergistic effect of the two compounds (α- and β-pinene), as well as their structural diversity in the active site of anticholinesterase, making it difficult to predict the potential for the structure-activity relationship.

### Cytotoxic activity

The oil of *P. aequale* was tested in vitro against three cancer cell lines (HCT-116, colon; ACP-03, gastric; SKMEL19, melanoma) with increasing concentrations (0.4–25.0 μg/ml) and compared with doxorubicin, a known anticancer drug, used in medical clinics. Only oils with IC_50_ values lower 25 μg/ml were considered active, in at least one cell line (Table 2). The oil was more potent against cell line of intestinal type gastric cancer (ACP-03, IC_50_ = 1.54 μg/ml), when compared with the cell line of colon cancer (HCT-116, IC_50_ = 8.69 μg/ml). The oil does not display effect against the cells of melanoma cancer (SKMEL19) when compared with the doxorubicin.

**Table 2:** Cytotoxic activity of the oil of *P. aequale* on cancer cell lines^a^

Sample		IC_50_ (μg/ml)^a^		Hemolysis
	HCT-116 (Colon)	SKMEL19 (Melanoma)	ACP-03 (Gastric)	(μg/ml)
Oil	8.69 (7.20–10.50)	> 25	1.54 (1.34–1.77)	> 200
Doxorubicin	0.10 μM (0.05–0.28)	0.3 μM (0.24–0.38)	0.27 μM (0.22–0.33)	> 200

^a^Data are presented as IC_50_ values and 95 % confidence intervals obtained by nonlinear regression for all cell lines from three independent experiments. Doxorubicin (DOX) was used as positive control. Only compounds with IC_50_ values lower than 25 μg/mL in at least one cell line were considered active

**Fig. 1:**
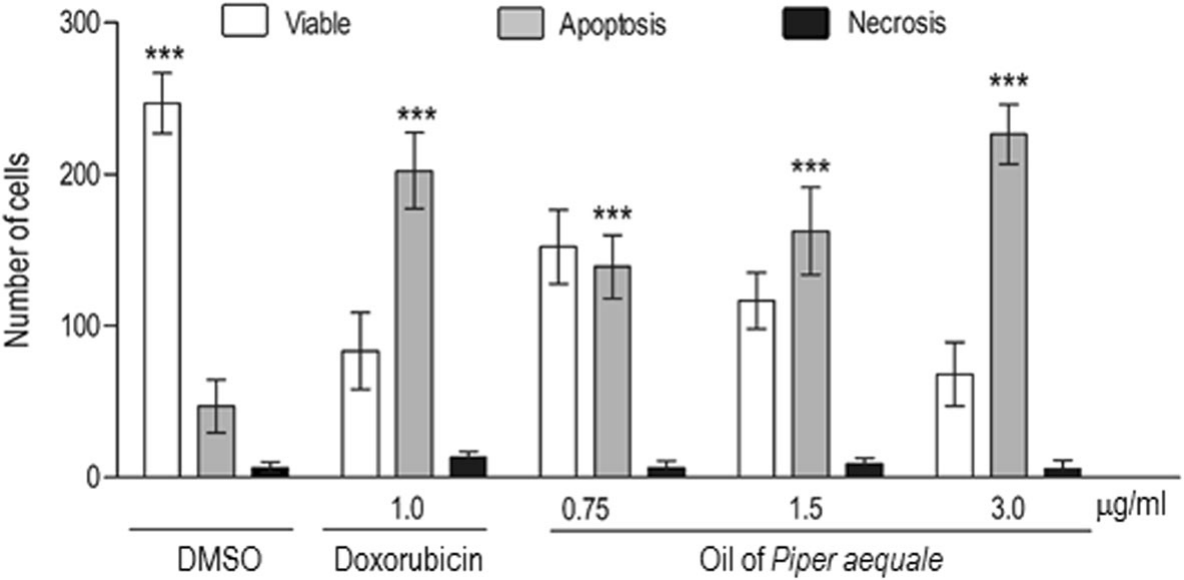
Cell death pattern by differential staining with ethidium bromide/acridine orange, after 72 h of treatment with the oil of *P. aequale.* Doxorubicin was used as a positive control. Bars represent the mean ± standard error of mean of three independent experiments. *** *P < 0.001* compared with the negative control by 2way ANOVA, followed by Bonferroni test

Also, the oil induces apoptosis in ACP-03 cell line, after 72 h of treatment, at all concentrations evaluated, when compared to negative control (*p* < 0.001). The oil at the concentration of 3.0 μg/ml did not display a significant difference in comparison to doxorubicin at 1.0 μg/ml. See Fig. 1.

In principle, the significant cytotoxic activity observed for the oil of *P. aequale*may be attributed to high content of δ-elemene (19.0 %), β-pinene (15.6 %) and α-pinene (12.6 %), probably, in association with the synergistic action involving other sesquiterpenes existing in the oil, as germacrene D, biciclogermacrene, β-atlantol and cubebol. Previously, the elemene-type sesquiterpenes were reported as proliferation inhibitors, stimulants of apoptosis and inductors of cell cycle arrest in the malignant cell [30–32]. Moreover, it has been found that β**-**elemene exerts anticancer activity of the brain, laryngeal, lung, breast, prostate, cervical, colon and ovarian [30, 33]. δ-elemene, an isomer with a different position at double bond existing in β-elemene, exerts antitumor activity by inducing apoptosis in cervical cancer cells, colorectal adenocarcinoma cells and human lung carcinoma mucoepidermoid cells [34–36].

## Conclusion

Based on the results, it can be assumed that the oil of *P. aequale* inhibits the proliferation by inducing the apoptosis of the human gastric cancer cells. This activity is probably related to a most global effect of δ-elemene, previously reported as a proliferation inhibitor, in association to other sesquiterpenes identified in the oil. Possible cytotoxic effect of oil was disregarded due to the absence of membrane damage. In this regard, the ability to induce lyses of mouse erythrocytes was unsuccessful.
